# All models are wrong and yours are useless: making clinical prediction models impactful for patients

**DOI:** 10.1038/s41698-024-00553-6

**Published:** 2024-02-28

**Authors:** Florian Markowetz

**Affiliations:** Cancer Research UK Cambridge Institute, University of Cambridge, Cambridge, UK

**Keywords:** Biomarkers, Mathematics and computing

*Most published clinical prediction models are never used in clinical practice and there is a huge gap between academic research and clinical implementation. Here, I propose ways for academic researchers to be proactive partners in improving clinical practice and to design models in ways that ultimately benefit patients*.

“All models are wrong, but some are useful” is an aphorism attributed to the statistician George Box. There is humility in claiming your model is wrong, but there is also bravado in implying your model might be useful. And, honestly, I don’t think it is. I think your model is useless. How would I know? I don’t even know who you are. Well, it is a bet. A bet I am willing to take because the odds are ridiculously in my favour.

I will explain what I mean in the context of clinical prediction models. My points apply to a wide range of preclinical models, both computational and biological, but my own core expertise is with clinical prediction tools. These are computational models from statistics, machine learning or AI that try to predict clinically relevant variables and ultimately aim to help doctors to treat patients better. The papers describing them make claims like “this model can be used in the clinic”; generally softened with words like “might”, “could”, “potential”, “promise”, or other techniques to reduce accountability. The Box quote offers a yardstick to measure the success of these models; not by how correctly they describe reality but by how useful they are in helping patients.

And in general, almost none of these tools ever help anyone. There is a wealth of systematic reviews in different fields to show how many models have been proposed and how few have even been validated, let alone been adopted in the clinic. For example, 408(!) models for chronic obstructive pulmonary disease were systematically reviewed^1^ and as a summary the authors bleakly note “several methodological pitfalls in their development and a low rate of external validation”. And whatever biomedical area you work in, your experiences will mirror this result – many novel prediction models, little help for patients. I believe that a model designed to be used for patients is useless unless it is actually used for patients.

## Lost in translation

To understand the challenges of translating academic research to the clinic and creating clinical decision tools that are actually being used, I don’t have to look further than my own papers. Some years ago my medical collaborators and I proposed a new classification of colon cancer into subtypes^2^ and helped to consolidate competing classification schemes^3^. To broaden applicability, we even designed histopathology markers for the subtypes^4^. This was a lot of work and we did our best to make the subtypes accessible and useful, but as far as I can tell, none of these classification schemes gets regularly used on patients. And the colon cancer subtypes are not an exception, the subtypes my lab helped to define in breast^5^ and pancreatic cancer^6^ are also not being widely used clinically.

By all academic standards our work was a success, it has been widely noted in the community, and the papers are well-cited. Still, it feels to me like there is something missing. Over the years I have come to see academic papers not as ends in themselves, but as the beginning of the journey to clinical implementation, and I am frustrated with how little of my own work ever had clinical impact. Looking back, here are some lessons I had to learn over the last two decades:

Observation 1: *Success in academia is not the same as success in the clinic.* Academic success is measured in papers, grants, impact factors and citations. The clinical success of your model is measured differently: How often is it being used in how many hospitals? And ultimately: how many patients does your model help? As my own examples above show, academic success does not necessarily lead to clinical success. Why? Because there is little incentive to actually implement an academic advance. Academic career rules prioritise novelty over implementation. As a result, fully engaging with translational research can slow your academic career, because publishing yet another model (no matter how useless) will help your career more than navigating the details of hospital software systems to implement an established model. This is particularly frustrating for junior researchers who need to “play the game” to make their next promotion. This systemic bias against implementation prevents benefits for patients.

Observation 2: *Successful models use data that are available in routine practice*. Not just the incentives, also the data differ between academia and the clinic. Large academic collections like TCGA^7^ make it look like integrating DNA with RNA with methylation with imaging with proteomics was already general practice - whereas in fact the only data you might have in clinical reality are an H&E slide and some DNA, hopefully from the same patient. As a result, the academic view of what constitutes an important step forward (more spatial! more multi-omics!) is at odds with clinical reality. This observation is particularly pertinent for gene expression data, which are academically ubiquitous^7–9^ but clinically have only led to a small number of success stories like OncotypeDx, MammaPrint or ProSigna to improve clinical decisions in breast cancer – disappointing for a field that was celebrated as a breakthrough more than 20 years ago^10^. Take Home message: If you make the wrong choice on what data to include into your model, you might kill your model’s usefulness before you even start training it.

Observation 3: *Successful models are linked to actions*. The reason the cancer subtyping studies I described above lack the impact I was hoping for is that they indicate differences in survival without being linked to a clear action. Some people do better and others do worse, so what? Similarly, the original PAM50 classifier for breast cancer subtypes^10^ had no action linked to it and was useless until the ProSigna test modified it into a prognostic score to recommend adjuvant chemotherapy to high-risk patients. What doctors really need to know is: What action should we take to help a particular patient? What drug, if any, should we give them? These are the questions your prediction tool needs to address to even have a chance of being useful. And the best way to find out if you are addressing an important clinical decision point is to engage closely with a wide variety of clinical practitioners and domain experts.

Observation 4: *Successful models are implemented outside of centres of excellence*. If you want to have impact, your tool needs to be used outside of Cambridge, Stanford or Zurich. Working with a research-savvy clinician at your home institution is necessary, but for real impact you need to reach out beyond your academic comfort zone and find out if doctors elsewhere find your tool useful, and how well it fits into different clinical pathways and decision procedures.

Observation 5: *Success in the clinic is hard earned*. A hospital’s responsibility towards their patients includes a duty to be receptive to innovation. But hospitals are conservative, highly regulated environments, where every change in established practices needs to be offset against potential harm to patients. Add to that the fact that health systems are underfunded and doctors are overworked, and you will see why you have to produce substantial evidence of the usefulness of your new approach before any hospital will even consider taking your academic insight on board. This can be very frustrating for academics who can feel that they have to go not one, but rather two or three extra miles to make their tool useful for patients.

## Mapping the path to usefulness

I know what you are thinking: “It takes time! Method development is trailblazing new ideas, the clinic will eventually catch up.” Yes, it will take time. But if “it takes time” is the only answer you have on how your model will be useful for patients, then it will take even longer.

Can we expect models to be useful immediately after publication? Probably not. But we can expect model developers to have a roadmap to the clinical usefulness of their model. For drug discovery, there is a well-known sequence of steps to follow^11^, and for medical software there are equally clear rules^12^, but they are in my experience much less well-known.

Many validations of your model, like clinical trials, can be done without regulatory approval. But establishing line of sight to the clinic early and planning a path through regulation will be crucial if you really want your model to be used on patients. Which regulation to follow will depend on your location. For example, in Europe it is CE marking, in the UK it is UKCA approval, in the US it is FDA approval, and other parts of the world have similar schemes. Regulators always seem scary, but in fact they want to help and the best advice is to reach out to them as early as possible.

It is a big task and needs to be broken down into more easily digestible chunks. Start outlining the road to implementation while you are still designing your clinical prediction tool: is it even theoretically possible that your model will help a patient? Is there a clear decision point your model is addressing with data that are clinically relevant? Continue solidifying your plan while writing a paper about your model: What exactly needs to happen for doctors to use your tool? What validations are needed? If you introduce a new data modality, how would this fit into existing infrastructure? And if the main use of your model is not for patients, but maybe hypothesis generation or testing a new modelling idea, then here is the place to state it clearly.

Including a section on implementation into the discussion of every translational paper would be the simplest and in my view most impactful step to improve current scientific practices. It will make your papers stronger and prepare you for your conversations with regulators.

Writing an implementation plan can only be a first step - actions are needed, not words - but spending more time thinking about implementation and regulation would make academic researchers much more proactive partners in improving clinical standards, and that would be a major step forward.

## Beyond academic success

Medical device implementation and regulation are rarely considered in academic practice, but are standards for start-ups and industry. In my own research, our work^13^ on AI models to analyse images taken by the Cytosponge, a minimally invasive alternative to endoscopy for detecting Barrett oesophagus, are the basis of Cyted, a company founded by one of my PhD students (www.cyted.ai). And our work on measuring different types of chromosomal instability^14^ led to the foundation of Tailor Bio, a genomics start-up with a pan-cancer precision platform (www.tailor.bio). Time will tell if these companies really deliver and translate our academic work widely into routine practice, but at least I am confident that they have a concrete and pragmatic plan on how to get there, because without it they would have never gotten funded.

And spin-outs are not the only way. One of the best examples I know of a successful medical decision tool is Predict Breast (https://breast.predict.nhs.uk/)^15^. This CE-marked model addresses an important medical question: how might different treatments for early invasive breast cancer improve survival rates after surgery. It only uses data almost all doctors have available (like age, HER2 status or tumour size). The user interface has been fine tuned by the Winton Centre for Risk and Evidence communication^16^. Predict Breast has been used by doctors world-wide 2.5 million times overall and 450,000 times in the past 12 months alone. This is the impact the rest of us only dream of! Predict Breast was never published in any high-impact journal or funded by a big grant; in fact, major funders triaged applications to fund it arguing that in their opinion Predict Breast was unlikely to ever be widely used. The costs for CE marking were covered by a philanthropic donation to the Winton Centre. This success story powerfully highlights that real-world impact is possible from within academia, but that it is impeded by current funding criteria.

When I asked Paul Pharoah, the lead developer of Predict Breast, what his secret to success was, he gave me a list of criteria for successfully building clinical prediction tools, which is the core of the list in Box 1. What you find in this list is a summary of all the requirements we have discussed in this text.

Following this checklist is what individual researchers can already do right now. In the future, a systemic shift towards implementation will need a concerted effort from university leaders, hospitals, journals and funders. University leaders need to prioritise implementation in their promotion criteria, hospitals need the resources to proactively engage with innovation, leading journals need to publish implementation successes rather than just novelty, and funders need to support these efforts by adapting the criteria for their grants and ensuring that expert panels have members with a proven track record in real-world implementation.

If you follow the checklist, your models will still be wrong. But they will finally have a chance to become useful.

Box 1 A checklist for useful clinical prediction tools

1. Do you address a clear clinical decision point?
2. Does your tool output parameters that help in that decision making?
3. Do you address a clear clinical decision point? Are you sure? Better go and talk to a clinical collaborator who is a domain expert.
4. Are the input parameters used in common clinical practice?
5. Do you address a clear clinical decision point? Are you really, really sure? Better go and get advice from a large and diverse group of experts and stakeholders.
6. Is the interface easy to use, both for input and output?
7. What value does your model add to current clinical judgement?
8. Is your tool better than existing tools?
9. What is your implementation plan?
  - What needs to happen for doctors to actually use this tool?
  - What is the path through medical device regulation?
  - Is the medical environment ready for it?
